# Effect of haemodialysis on the brain and heart assessed using multiparametric MRI

**DOI:** 10.1093/ndt/gfaf117

**Published:** 2025-07-09

**Authors:** Eleanor F Cox, Venkata Rukmini Latha Gullapudi, Charlotte E Buchanan, Kelly White, Rosemary Nicholas, Bernard Canaud, Maarten W Taal, Nicholas M Selby, Susan T Francis

**Affiliations:** Sir Peter Mansfield Imaging Centre, School of Physics & Astronomy, University of Nottingham, Nottingham, UK; NIHR Nottingham Biomedical Research Centre, Nottingham University Hospitals NHS Trust and the University of Nottingham, Nottingham, UK; Centre for Kidney Research and Innovation, University of Nottingham, Derby, UK; Renal Unit, University Hospitals of Derby and Burton NHS Foundation Trust, Derby, UK; Sir Peter Mansfield Imaging Centre, School of Physics & Astronomy, University of Nottingham, Nottingham, UK; Renal Unit, University Hospitals of Derby and Burton NHS Foundation Trust, Derby, UK; Sir Peter Mansfield Imaging Centre, School of Physics & Astronomy, University of Nottingham, Nottingham, UK; Fresenius Medical Care Deutschland GmbH, Germany; Centre for Kidney Research and Innovation, University of Nottingham, Derby, UK; Renal Unit, University Hospitals of Derby and Burton NHS Foundation Trust, Derby, UK; Centre for Kidney Research and Innovation, University of Nottingham, Derby, UK; Renal Unit, University Hospitals of Derby and Burton NHS Foundation Trust, Derby, UK; Sir Peter Mansfield Imaging Centre, School of Physics & Astronomy, University of Nottingham, Nottingham, UK; NIHR Nottingham Biomedical Research Centre, Nottingham University Hospitals NHS Trust and the University of Nottingham, Nottingham, UK

**Keywords:** brain ageing, brain swelling, ESRD, haemodialysis, multiparametric magnetic resonance imaging

## Abstract

**Background and hypothesis:**

Haemodialysis (HD) patients often develop cognitive impairment, negatively impacting health-related quality of life. We use brain magnetic resonance imaging (MRI) measures to study the acute changes in cerebral water content during HD, alongside chronic changes in HD patients compared with healthy volunteers (HVs) to assess whether the brain changes associated with ageing develop more rapidly in HD patients (‘accelerated brain ageing’). We also study associated cardiac MRI measures.

**Methods:**

3T MRI scans were performed during HD in 12 patients to characterize the acute effect of HD on cerebral water content (T_1_ mapping), alongside previously reported results from the HD-REMODEL (HaemoDialysis interventions to REduce MultiOrgan Dysfunction and Effect on quality of Life assessed by MRI scanning) trial. MRI changes in brain structure [volumes and T_1_ of white (WM) and grey matter (GM), WM diffusion fractional anisotropy (FA) and mean diffusivity (MD)], perfusion, blood flow, and cardiac measures were compared between HD patients pre-dialysis and HVs (age and gender matched).

**Results:**

WM T_1_ increased during HD (3.8 ± 1.7%, *P* = .0005). GM and WM volume [total intracranial volume (TIV)-corrected] were lower in HD compared with HVs [GM volume/TIV: 0.37 (0.34–0.41) vs 0.42 (0.42–0.44), WM volume/TIV: 0.34 ± 0.03 vs 0.37 ± 0.01, *P* = .009]. In HD, FA was lower and MD higher than HVs (FA: 0.32 ± 0.02 vs 0.35 ± 0.01, MD: 0.59 ± 0.03 vs 0.53 ± 0.01, *P* < .0001). Higher MD and lower FA was seen in older participants, with steeper slopes in HD (MD: 0.003 vs 0.0006 × 10^−3^ mm^2^/s/year *P* = .003, FA: –0.001 vs –0.0003 units/year *P* < .0001), suggestive of accelerated ageing. There were no differences between groups in age-related heart changes.

**Conclusions:**

An acute increase in WM T_1_ during HD has been shown for the first time, reflecting a rise in brain water content. This is potentially caused by the development of an osmotic gradient across the blood–brain barrier due to slower diffusion of urea, and may contribute to acute symptoms and chronic pathological changes contributing to accelerated brain ageing in HD patients.

## Graphical Abstract

**Graphical Abstract:**
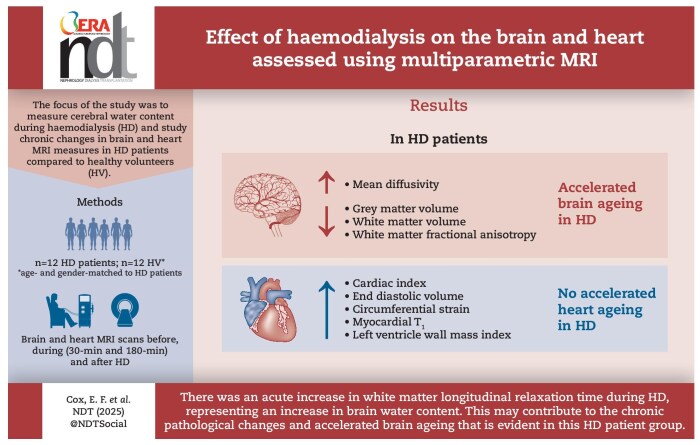

KEY LEARNING POINTS **What was known:**

- Cognitive impairment is common in people receiving haemodialysis (HD) and negatively impacts health-related quality of life.
- Multiple HD-related mechanisms are likely to accelerate brain ageing.
- Although changes in brain volume have been previously reported in response to dialysis, the impact of osmotic fluid shifts during dialysis on the brain have not previously been studied in detail.

**This study adds:**

- The study of brain and cardiac changes that occur during dialysis in a unique way using magnetic resonance (MR) imaging.
- By measuring the intra-dialytic change in the MR longitudinal relaxation time (T_1_) for the first time, we demonstrate a significant increase in white matter T_1_ during HD that indicates an acute rise in brain water content.
- Comparison of pre-dialysis scans with those of healthy volunteers shows evidence of accelerated brain ageing, but not accelerated ageing of the heart.

**Potential impact:**

- An increase in brain water content during HD may contribute to accelerated brain ageing resulting in cognitive impairment, as well as to acute symptoms immediately following dialysis.
- Modifications to the HD procedure that reduce the rate of osmotic flux should be investigated to mitigate these effects.

## INTRODUCTION

Patients with kidney failure receiving maintenance dialysis are at increased risk of cognitive impairment and dementia, with up to two-thirds displaying moderate to severe deficits in cognitive function [1–3]. This may significantly impact functional status and the ability to perform activities of daily living and negatively impacts health-related quality of life (HR-QoL), hospitalization rates and survival [4]. A reduction in cognitive function occurs as people age (brain ageing) and is associated with a range of changes including brain structure and function. ‘Accelerated brain ageing’ refers to structural or functional brain changes that occur at a faster rate than expected for a person's chronological age, and can occur due to lifestyle, environmental and genetic factors, as well as disease. Abnormalities have been shown to occur in the brain of dialysis patients using magnetic resonance imaging (MRI), including grey (GM) and white matter (WM) atrophy, cerebral microbleeds, subclinical infarcts, periventricular WM disease (leukoaraiosis), reduced WM structural integrity [6, 7] and changes in resting-state functional MRI networks [7].

Efforts to understand the mechanisms that underlie the development of such pathologies have included the study of acute changes in the brain following haemodialysis (HD) treatments [8]. Studies have reported that dialysis can lead to brain swelling (increased brain volume) attributed to osmotic fluid shifts [9, 10], acute changes in WM structural integrity [10, 11] and reduced cerebral perfusion [12]. In the HD-REMODEL trial (HaemoDialysis interventions to REduce MultiOrgan Dysfunction and Effect on quality of Life assessed by MRI scanning), a randomized cross-over study of standard versus thermocontrolled HD, we demonstrated dialysis-induced reductions in carotid and basilar artery blood flow using intradialytic MRI [13]. These changes occurred in conjunction with significant reductions in cardiac output and left ventricular contractility, although we did not observe a significant change in GM perfusion. This finding suggests that non-haemodynamic processes may occur concurrently during dialysis, and further research is required to delineate these mechanisms and their relative importance to the development of chronic brain and cardiac pathology.

Here we perform a secondary analysis of the HD-REMODEL trial. Our primary focus is on previously unreported changes in cerebral water content (inferred from T_1_ mapping [14]) that occur during HD as a process that may occur independently from changes in brain perfusion and cardiac output. Secondly, we hypothesize that chronic changes occur in brain and cardiac MRI measures in HD patients compared with healthy volunteers with equivalent age and gender.

## MATERIALS AND METHODS

This is secondary analysis of the HD-REMODEL trial (ClinicalTrials.gov Identifier: NCT03280901) [13]. Specifically, we study the additional T_1_ MRI brain measure collected during dialysis to characterize the acute effect of HD on cerebral water content, alongside previously reported results of brain perfusion, large vessel flow and cardiac output. Here we report data from the standard HD arm. As there were no differences in any intradialytic MRI measures between the standard and thermocontrolled arms of the HD-REMODEL trial [13], any missing data points in the standard arm were imputed using corresponding values from the thermocontrolled arm [13].

### Participants

Participants in the HD-REMODEL trial (aged 48–77 years) had been receiving HD for kidney failure via an arteriovenous fistula for >3 months. Exclusion criteria included hypotension during HD in the 4 weeks prior to recruitment or New York Heart Association Stage IV heart failure (to ensure stable participants taking part in the MRI study in order to minimize the risk of adverse events), active infection or malignancy or contraindications to MRI.

For this analysis, we additionally recruited a one-to-one age- (±5 years) and gender-matched group of healthy volunteers (HVs) to match patients who completed at least one session of intradialytic MRI. This group had no known heart, kidney or neurodegenerative disease; diseases affecting brain morphometry (e.g. multiple sclerosis, epilepsy, stroke, diabetes, Parkinson's disease); subjective memory impairment; history of depression; alcohol misuse; or contraindications to MRI.

The HD-REMODEL trial was approved by an Independent Research Ethics Committee (East Midlands Research Ethics Committee, reference 17/EM/0235), and the HV study was approved by the University of Nottingham Research Ethics Committee 166-1812). All participants provided written informed consent.

### Study procedure

HD-REMODEL was a randomized, open-label, blinded endpoint, crossover trial [13] where participants were randomly allocated to receive either standard or thermocontrolled HD for 2 weeks before undergoing serial MRI scan sessions at four time points: pre-dialysis, during dialysis (commencing at 30-min and 180-min after the start of dialysis) and post-dialysis. Participants then crossed to the other modality to repeat the study protocol.

Dialysis sessions were conducted using a Fresenius 5008 machine at the Sir Peter Mansfield Imaging Centre (University of Nottingham), with adapted facilities to enable intradialytic MRI [13, 14]. The dialysis machine was situated immediately outside the scan room, with bloodlines directed through waveguides and connected to non-ferrous 15 G silicon dialysis needles to cannulate the arteriovenous fistula. Bloodlines required extension by 0.6 m on both arterial and venous sides. Dialysate flow was 500 mL/min, minimum blood pump speed 300 mL/min. The participant's usual anticoagulation was used, and dialysate composition was sodium 137 mmol/L, potassium 2.0 mmol/L, calcium 1.25 mmol/L, magnesium 0.5 mmol/L and glucose 1.0 g/L.

Routine clinical data and demographic details of the HD patients were collected from medical records. Blood pressure (BP) was recorded every 15 min during dialysis. Clinical measurements taken before and after dialysis included body weight and ultrafiltration volume, and standard haematology and biochemistry blood tests. Cognitive assessments included the Montreal Cognitive Assessment (MoCA) for use as a brief cognitive screening tool to assess mild cognitive impairment [16], and the Trail Making Test (TMT) parts A and B [17] to provide an index on cognitive flexibility. These assessments were collected pre- and post- dialysis; further details are provided in the Supplementary Methods.

### MRI acquisition and analysis

MRI measures were collected on a 3.0 Tesla Ingenia wide-bore scanner (Philips Healthcare Systems, Best, The Netherlands) using a Torso coil combined with posterior coil and HeadSpine coil. Prior to dialysis, HD participants underwent an 80-min brain and cardiac MRI protocol (Scan 1). They then had a 60-min brain and cardiac MRI scan session during (Scans 2 and 3) and post-dialysis (Scan 4) during which cardiac scans were first collected followed by brain scans. HVs underwent the 80-min Scan 1 protocol only. The MRI acquisition and measured parameters are outlined below, with further details provided in the Supplementary Methods.

#### Brain volume and white matter tracts (Scan 1 only)

GM, WM and cerebrospinal fluid (CSF) volume were assessed using a 3D magnetization-prepared rapid acquisition with gradient echo (MPRAGE) sequence. MPRAGE data were segmented into GM, WM and CSF tissue classes, and the volume of each computed and corrected for total intracranial volume (TIV).

A spin-echo EPI (SE-EPI) diffusion tensor imaging (DTI) sequence was used to assess WM tracts from fractional anisotropy (FA), the degree of anisotropy of a diffusion process, and mean diffusivity (MD), the magnitude of water diffusion within brain tissue.

#### Brain haemodynamics and water content assessed by T_1_ relaxometry (Scans 1–4)

GM perfusion was measured using Arterial Spin Labelling (ASL) data acquired with a FAIR labelling scheme and SE-EPI readout. A base equilibrium M_0_ scan with no labelling and inflow scan were also collected for perfusion quantification [18]. An SE-EPI T_1_ mapping scheme was collected using an inversion recovery acquisition. Partial volume (PV) maps of GM, WM and CSF were created from T_1_ maps and used to estimate GM perfusion, and GM, WM and CSF T_1_.

Cerebral blood flow of the right and left internal carotid artery flow and basilar artery flow was assessed using phase-contrast MRI (PC-MRI) and the mean vessel cross-sectional area, blood velocity and blood flow through the internal carotid and basilar arteries computed.

#### Cardiac structure and function (Scans 1–4)

Cardiac T_1_ mapping was collected using a 5(3)3 cardiac-triggered modified Look-Locker inversion recovery (MOLLI) scheme through the left ventricle (LV) short axis. LV wall mass, cardiac output, stroke volume and end diastolic volume (EDV) were assessed from a short axis (SA) cine and adjusted for body surface area to give LV wall mass index, cardiac index (CI), stroke volume index and indexed EDV. Ejection fraction was also calculated. Myocardial wall strain (longitudinal and circumferential) was assessed using MR tagging of the LV.

### Statistical analysis

Statistical analysis was performed using GraphPad Prism version 10.3.0(507) (GraphPad Software Inc., La Jolla, CA, USA), with *P* < .05 considered statistically significant. Normality was assessed with Shapiro–Wilk test.

Intradialytic effects were assessed using mixed-effects analysis (the Geisser–Greenhouse correction for unequal variability of differences) since repeated measures analysis of variance (RM-ANOVA) cannot handle missing values. This mixed model uses a compound symmetry covariance matrix and is fit using Restricted Maximum Likelihood. In the absence of missing values, this method gives the same *P*-values and multiple comparisons tests as RM-ANOVA. In the presence of missing values (missing completely at random), results are interpreted like RM-ANOVA.

Comparisons between the HD and HV groups were made with an unpaired *t*-test if both groups were normally distributed, otherwise a Mann–Whitney test was performed. Grouped data are expressed as mean ± standard deviation if both groups were normally distributed, otherwise grouped data are expressed as median (interquartile range).

The effects of age and dialysis vintage on MR parameters in the HD patients were assessed with multiple linear regression. Associations of MR parameters with dialysis vintage were assessed in the HD group with simple linear regression. The relationships of MR parameters with age were assessed with a simple linear regression for both groups and regression slopes compared between groups. ‘Accelerated brain ageing’ was inferred if the regression slopes for a given parameter (i.e. rate of change with age) were significantly different between the HD and HV groups. Due to technical difficulties, DTI data were acquired in only four HV subjects, therefore we inputted 11 additional HV datasets (49–76 years) collected with an identical acquisition (see Supplementary Material) who were not part of the original HV group.

## RESULTS

Twelve HD patients completed at least one intradialytic MRI scan session and were compared with 12 age- and gender-matched HVs. Demographics and clinical characteristics are provided in Table 1. As reported previously, systolic BP and diastolic BP decreased significantly during dialysis treatment but recovered by the end of treatment [13].

**Table 1:** Demographics, clinical characteristics and lab variables.

Parameter	HVs (*n* = 12)	HD (*n* = 12)
Age (years)	64 (56–72)	60 (51–76)
Male, *n* (%)	10 (83)	10 (83)
BMI (kg/m^2^)	27 ± 3	27 ± 4
Weight (kg)	86 ± 13	80 ± 15
eGFR >60 mL/min/1.73 m^2^, *n* (%)	11 (92)	0
Taking antihypertensive medications, *n* (%)	4 (33)	7 (58)
Aetiology of kidney disease, *n* (%)		
Diabetes		3 (25)
Glomerulonephritis		3 (25)
Hypertension/ischaemic nephropathy		2 (17)
Chronic pyelonephritis		2 (17)
Interstitial nephritis		1 (8)
Polycystic nephropathy		0
Unknown		1 (8)
Comorbid conditions, *n* (%)		
Ischaemic heart disease		1 (8)
Cerebrovascular disease		1 (8)
Hypertension		11 (92)
Liver disease		1 (8)
Ischaemic/neuropathic ulcers		1 (8)
Previous fracture		2 (17)
Time since dialysis initiation (months)		20 (5–56)
Ultrafiltration volume (mL)		2117 ± 753
Bioimpedance		
Total body water (L)	43 ± 8	40 ± 5
Intracellular water (L)	24 ± 4	21 ± 3
Extracellular water (L)	20 ± 4	19 ± 2
Over hydration (L)	0.07 ± 2.0	1.5 ± 1.5
Blood test results		
Haemoglobin (g/L)	145 ± 13	117 ± 9
Haematocrit (%)	42 ± 4	36 ± 3
Sodium (mmol/L)	142 (139–142)	140 (138–142)
Potassium (mmol/L)	4.1 ± 0.4	5.7 ± 1.0
Urea (mmol/L)	5.0 ± 1.3	21.6 ± 5.6
Creatinine (μmol/L)	81 ± 17	731 ± 260

Data are presented as mean ± standard deviation, median (interquartile range) or *n* (%).

BMI, body mass index; eGFR, estimated glomerular filtration rate.

Cognitive assessment results, shown in Table 2, are within the normal range for cognitive functioning.

**Table 2:** Cognitive test results in HD participants and HVs.

		HD (*n* = 11)		*P*-value	
Cognitive test	HVs (*n* = 12)	Pre-dialysis	Post-dialysis	HVs vs HD pre-dialysis	HD pre- vs post-dialysis
MoCA score	28.5 (27.3–29.0)	28.0 (26.0–28.0)	28.0 (27.0–29.0)	.07	.4
TMT Part A completion time (s)	24 (17–30)	30 (25–35)	30 (21–44)	.1	.9
TMT Part B completion time (s)	53 (35–71)	51 (39–77)	59 (49–85)	.6	.9

### Acute effects of HD on brain T_1_ relaxometry water content and haemodynamics

**Figure 1:**
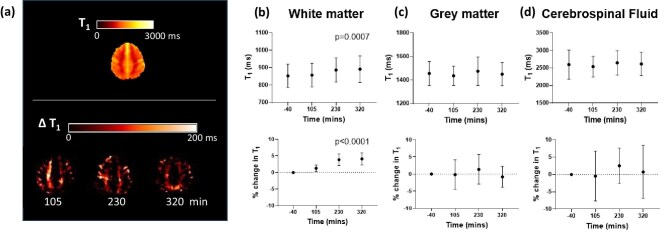
Acute effect of dialysis on GM and WM T_1_. (**a**) Averaged T_1_ map across all patients pre-dialysis, with the change from pre-dialysis in T_1_ on subsequent scans. Absolute T_1_ and percentage change in T_1_ across each time point for (**b**) WM, (**c**) GM and (**d**) CSF. The *x*-axis indicates the time at which the brain T_1_ mapping measurement was collected after the start of dialysis.

Figure 1a shows a mean T_1_ map of HD patients pre-dialysis, and the T_1_ as a function of the time this scan was collected after the start of dialysis and the % change in T_1_. There was a significant increase in WM T_1_ (3.8 ± 1.7%) from Scan 1 to Scan 3 [818 (802–901) ms vs 852 (835–951) ms, *P* = .0005] which persisted at Scan 4 post-dialysis (Fig. 1b). There were no changes in GM or CSF T_1_ during dialysis (Fig. 1c and d). As previously reported [13], GM perfusion did not change during dialysis, but there was a reduction in left carotid artery and basilar artery velocity flow.

### Comparison of brain MRI measures between HD and HVs

**Figure 2:**
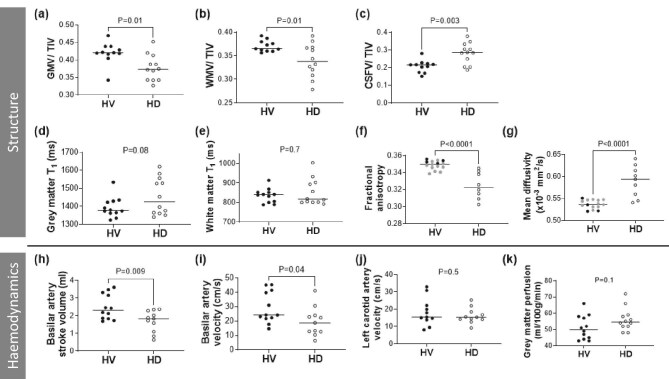
Comparison of brain MR measures of structure and haemodynamics between 12 HD patients and 12 age-matched HVs. Filled grey circles indicate additional data from HVs acquired with an identical acquisition. The MR measures shown previously in the HD group to decrease during dialysis (as reported in [13]) were basilar artery velocity and left carotid artery velocity, with no change in GM perfusion. Note not all measures were collected during dialysis. GMV, GM volume; WMV, WM volume; CSFV, CSF volume.

Table 3 compares brain MRI measures between HD and HV groups. TIV-corrected GM and WM volume were significantly lower in the HD group compared with HVs (Fig. 2a and b) with a proportionally larger CSF volume (Fig. 2c). In the HD group, the lower WM volume was widespread, whilst the reduced GM volume was localized to the right middle frontal gyrus. No brain regions showed higher WM or GM volume in the HD group compared with the HV group.

**Table 3:** Cerebral MRI measures: comparison of HD participants and HVs.

	HVs		HD			
Parameter		*n*		*n*	*P*-value	Effect size Difference [HD – HV] (95% confidence interval)
TIV (mL)	1463 ± 135	11	1393 ± 135	12	.2	
GMV/TIV	0.42 (0.42–0.44)	11	0.38 ± 0.04	12	.007	–0.04 (–0.07 to –0.01)
WMV/TIV	0.37 ± 0.01	11	0.34 ± 0.04	12	.01	–0.03 (–0.05 to –0.01)
CSFV/TIV	0.21 ± 0.03	11	0.28 ± 0.06	12	.002	0.07 (0.03 to 0.11)
FA WM	0.35 ± 0.01	15	0.32 ± 0.02	9	<.0001	–0.03 (–0.04 to –0.02)
MD WM (×10^−^^3^ mm^2^/s)	0.53 ± 0.01	15	0.59 ± 0.04	9	<.0001	0.05 (0.03 to 0.07)
T_1_ GM (ms)	1394 ± 56	12	1455 ± 102	12	.08	
T_1_ WM (ms)	841 (802–853)	12	818 (802–901)	12	.7	
T_1_ CSF (ms)	2818 ± 410	12	2594 ± 413	12	.2	
Basilar artery stroke volume (mL)	2.5 ± 0.7	12	1.7 ± 0.6	11	.009	–0.8 (–1.4 to –0.2)
Basilar artery flux (mL/s)	2.5 ± 0.7	12	1.9 ± 0.8	11	.07	
Basilar artery area (mm^2^)	8.2 (7.7–11.2)	12	10.5 (7.3–11.7)	11	.4	
Basilar artery velocity (cm/s)	29 ± 11	12	20 ± 10	11	.04	–10 (–19 to 0)
Right carotid artery stroke volume (mL)	3.0 ± 1.8	11	3.5 ± 1.4	11	.5	
Left carotid artery stroke volume (mL)	3.6 ± 2.0	11	3.3 ± 1.5	11	.7	
Right carotid artery flux (mL/s)	3.1 ± 1.7	11	3.8 ± 1.6	11	.3	
Left carotid artery flux (mL/s)	3.6 ± 2.0	11	3.6 ± 1.6	11	>.9	
Right carotid artery area (mm^2^)	19.0 ± 6.4	11	22.9 ± 9.2	11	.3	
Left carotid artery area (mm^2^)	19.8 ± 9.2	11	22.3 ± 8.0	11	.5	
Right carotid artery velocity (cm/s)	16.1 ± 6.4	11	17.4 ± 6.2	11	.6	
Left carotid artery velocity (cm/s)	18.2 ± 7.9	11	16.2 ± 4.4	11	.5	
Perfusion GM (threshold = 0.75) (mL/100 g/min)	51 ± 7	12	56 ± 7	12	.1	

Data are presented as mean ± standard deviation or median (interquartile range).

GMV, GM volume; WMV, WM volume; CSFV, CSF volume.

There was no difference in WM T_1_ between groups, albeit with a trend towards higher GM T_1_ in the HD group (Fig. 2d and e). In the HD group, the DTI metric FA was lower and MD higher compared with the HV group (Fig. 2f and g).

Basilar artery stroke volume and velocity were both lower in the HD group compared with the HV group (Fig. 2h and i). There were no differences between groups for carotid artery measures or GM perfusion (Fig. 2j and k).

### Accelerated brain ageing in HD participants

**Figure 3:**
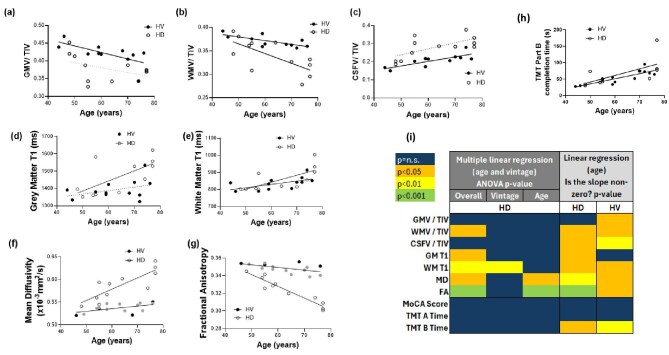
Correlation of (**a**–**g**) brain MR measures of structure and water content and (**h**) TMT part B completion time with age. Solid regression lines indicate a significant correlation, dotted regression lines are not significant. Filled grey circles indicate additional data from healthy subjects acquired with an identical acquisition. (**i**) *P*-values from linear regression of brain MRI measures of structure and water content and cognitive assessment results with age for the HD and HV groups, with and without using dialysis vintage as a covariate in the HD group. GMV, GM volume; WMV, WM volume; CSFV, CSF volume.

Figure 3 shows regression of brain MRI measures and cognitive assessment with age for both the HD and HV groups, and with dialysis vintage as a covariate in the HD group. Full regression parameters are given in Supplementary data, Table S1. In the HV group, TIV-corrected GM and WM volumes were lower (Fig. 3b and h), whilst TIV-corrected CSF volume was higher in older participants (Fig. 3c and h). In the HD group, TIV-corrected WM volume was lower in older participants, with no effect of dialysis vintage, with a trend for a steeper slope in the HD group compared with HV group (Fig. 3b, *P* = .09).

GM T_1_ did not vary with age in the HV group (Fig. 3d and h), although WM T_1_ positively correlated with increasing age (Fig. 3e and h). In the HD group, WM T_1_ was more influenced by dialysis vintage than age. No other direct associations were found between MR parameters and dialysis vintage.

In the HD group, DTI metrics changed primarily with age and with no effect of dialysis vintage, with increased MD and decreased FA with ageing (Fig. 3f–h). The same trends with ageing were seen in the HV group but the slopes were significantly different between groups. The rate of change of MD was 0.0006 × 10^−3^ mm^2^/s per year for HVs and 0.003 × 10^−3^ mm^2^/s per year for HD (*P* = .003) and for FA, the rate of change was –0.0003 units per year for HVs and –0.001 units per year for HD (*P* < .0001), indicating a faster change with age in the HD group for both DTI metrics. Haemodynamic measures of perfusion and brain blood flow did not significantly vary with age or dialysis vintage in either the HD or HV group. Regression of the cognitive assessment results with age showed an increase in TMT part B completion time for HD and HV groups. This was no longer significant in the HD group once dialysis vintage was included in the regression.

### Cardiac MRI measures between HD and HV groups and associations with ageing

**Figure 4:**
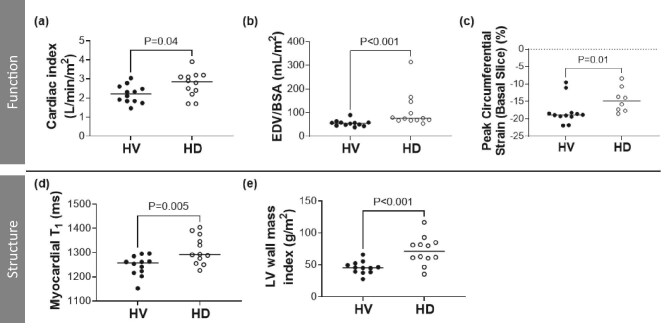
Comparison of heart MR measures of structure and function between 12 HD patients and 12 age-matched HVs. The MR measures shown previously in the HD group to decrease during dialysis (as reported in [13]) were cardiac index and peak circumferential strain, with no change in myocardial T_1_ or LV wall mass index. Note not all measures were collected during dialysis. BSA, body surface area.

Table 4 compares cardiac MRI measures between groups. In the HD group, CI, indexed EDV, myocardial T_1_ and LV wall mass index were higher than in the HV group (Fig. 4a, b, d and e) and circumferential strain less negative (less contractile) (Fig. 4c). No other cardiac MR measures were different between groups.

**Table 4:** Cardiac MRI measures: comparison of HD participants and HVs.

	HVs		HD			
Parameter		*n*		*n*	*P*-value	Effect size Difference [HD – HV] (95% confidence interval)
Heart rate (bpm)	64 ± 7	12	69 ± 10	12	.2	
Heart rate variability (bpm)	14 (7–25)	12	9 (6–11)	12	.08	
Ejection fraction (%)	65 ± 5	12	63 ± 8	12	.5	
Stroke volume index (mL/m^2^)	34 (32–40)	12	40 (32–48)	12	.2	
Cardiac index (L/min/m^2^)	2.2 ± 0.5	12	2.7 ± 0.7	12	.04	0.5 (0.0 to 1.0)
LV EDV/BSA (mL/m^2^)	55 (46–58)	12	76 (67–134)	12	.0003	21 (12 to 55)
Myocardial T1 (ms)	1246 ± 42	12	1311 ± 58	12	.005	65 (22 to 108)
Strain						
LA (%)	–15 ± 4	12	–14 ± 3	8	.6	
SA apical slice (%)	–22 ± 3	11	–21 ± 5	8	.7	
SA mid-slice (%)	–20 ± 2	10	–19 ± 5	9	.4	
SA basal slice (%)	–19 (–19 to –18)	12	–16 (–18 to –14)	8	.01	4 (1 to 8)
LV wall mass index (g/m^2^)	46 ± 10	12	72 ± 22	12	.0009	26 (12 to 41)

Data are presented as mean ± standard deviation or median (interquartile range).

bpm, beats per minute; BSA, body surface area; LA, longitudinal axis; SA, short axis.

**Figure 5:**
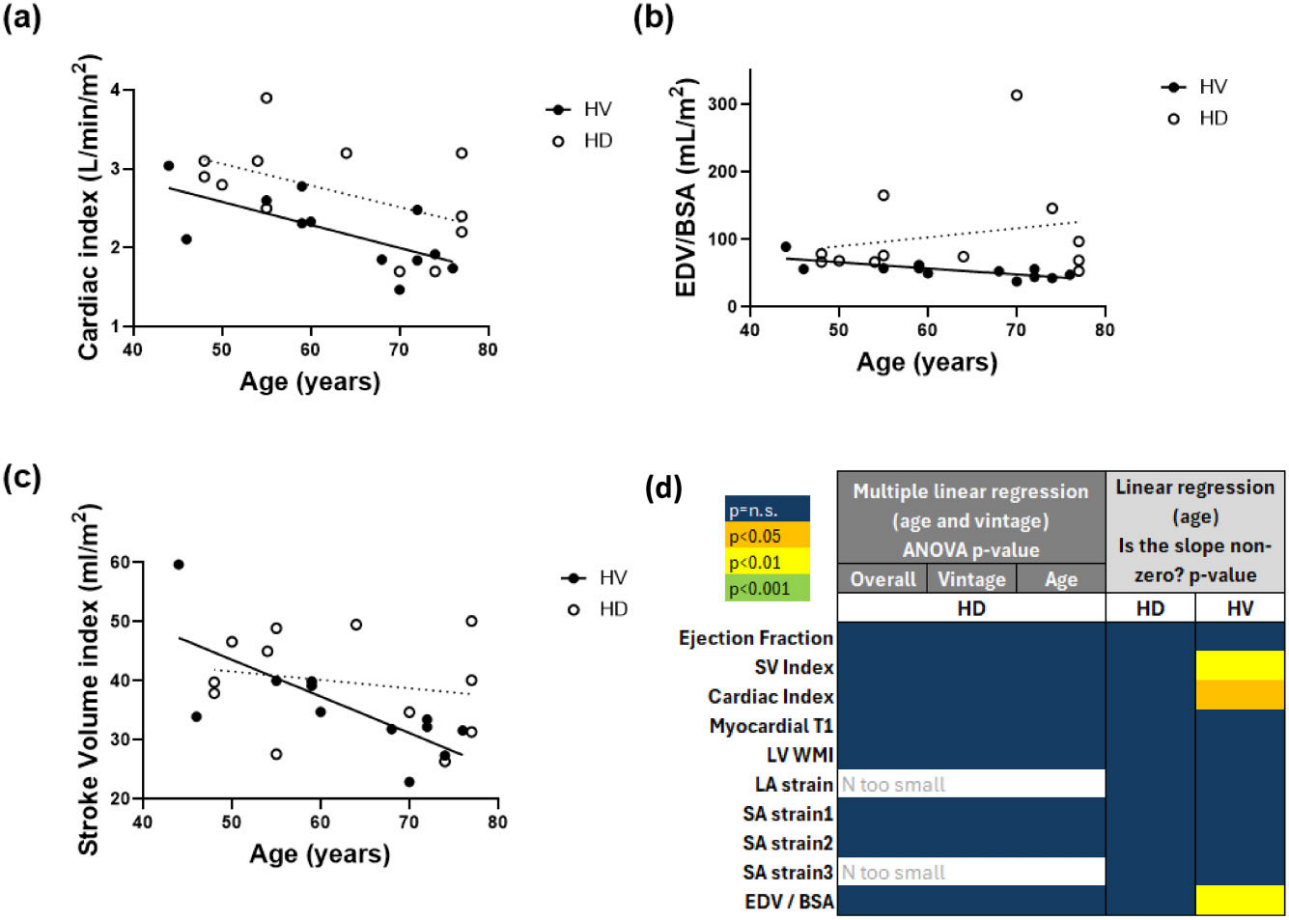
(**a**–**c**) Correlation of heart MRI measures of structure and function with age and vintage. Solid regression lines indicate a significant correlation, dotted regression lines are not significant. (**d**) *P*-values from linear regression of heart MRI measures of structure and function with age for the HD and HV groups, with and without using dialysis vintage as a covariate in the HD group. BSA, body surface area; SV, stroke volume; WMI, wall mass index; LA, long axis; SA, short axis.

Figure 5 shows regression of cardiac MRI measures with age for both the HD and HV groups, and with dialysis vintage as a covariate in the HD group. Full regression parameters are given in Supplementary data, Table S2. CI, stroke volume index and indexed EDV decreased with age in the HV group, but there were no associations with age or dialysis vintage in the HD patients. As previously reported [13], CI decreased during dialysis, and peak longitudinal and circumferential strain became less negative during dialysis.

## DISCUSSION

We assessed the acute effects of HD on the brain using intradialytic MRI and show for the first time an acute increase in WM T_1_ by the third hour of dialysis suggesting a rise in WM tissue water content, potentially due to the development of an osmotic gradient across the blood–brain barrier because of the relatively slow diffusion of organic osmolytes like urea. This is likely to be relevant to symptoms that some patients notice after dialysis (e.g. brain ‘fog’). In addition, we quantify chronic brain changes in dialysis patients compared with age-matched healthy volunteers using structural, DTI, blood flow and perfusion MRI measures. Several of the abnormalities in brain structure and structural integrity are consistent with accelerated brain ageing in dialysis patients which is not seen in the cardiac MRI measures.

We observed an intradialytic increase in WM T_1_ which persisted post-dialysis, suggesting an acute increase in WM water content [19] during dialysis. This is supported by studies which have reported an increase in brain volume during dialysis [9–11]. The most extreme manifestation of brain swelling during dialysis is disequilibrium syndrome [20], but even in patients without overt symptoms, MRI studies measuring brain structure have demonstrated acute increases in brain volume after dialysis. This has been attributed to cerebral oedema resulting partly from slower diffusion of urea across the blood–brain barrier during dialysis and the resulting increase in brain-to-plasma urea ratio favouring movement of water into the brain tissue [21]. More recently, cytotoxic oedema caused by subclinical ischaemia has been suggested as an alternative mechanism. Anazodo *et al*. [11] found WM and GM volume increased between pre-dialysis and 60 min before the end of dialysis and concluded that the predominant pattern of acute increases in FA and reductions in diffusivity were suggestive of ischaemic insults sustained during dialysis. Schaier *et al.* [10] observed acute changes in WM integrity with DTI metrics compared between pre- and post-dialysis measures, but conversely reported reduced FA and increased diffusivity alongside increases in GM and WM volumes within specific anatomical clusters. Differences in the relative contribution of pathophysiological processes between individuals could in part explain inconsistent results between these studies in HD participants. We did not observe an intradialytic change in GM T_1_, although previous studies report increased GM volume following dialysis [9–11]. Our observations indicate that increases in brain volume during dialysis are at least in part due to an increase in WM water content resulting from the osmotic effects. We can speculate about whether there are differential effects between anatomical regions with greater vulnerability seen in WM, or that a lack of change in GM perfusion in our HD group avoided the development of cytotoxic oedema.

Our results confirm significant chronic brain abnormalities in patients receiving maintenance dialysis compared with healthy participants. These include reduced GM and WM volumes, with GM reduction focussed in the right middle frontal gyrus, an important area for numeracy skills, and part of the multiple demand system [22]. T_1_ and perfusion were similar between the HD and HV groups, but there were significant differences in DTI metrics. Intact myelinated fibres restrict the movement of water within and across fibre bundles, but pathological changes that affect WM integrity (e.g. dysmyelination, glial-induced inflammation, dysfunctional extracellular debris, axonal degeneration, ischaemic injury) lead to increased water diffusivity [23]. Consistent with this, we observed lower FA and higher MD in HD patients, a pattern commonly seen in ageing [24], and consistent with previous studies in dialysis patients [25].

Whilst many MRI measures were associated with age in both HV and HD groups, it was notable that there were steeper age-related changes in the HD group, particularly in WM volume and DTI metrics. These results indicate a pattern of accelerated brain ageing in HD patients, consistent with higher incidence and more rapid progression of cognitive impairment in this group [26]. This was despite the cognitive assessment MoCA and TMT results for our HD participants pre-dialysis being within a normal range.

Our HV data followed the same trends with age as cardiac measures in the UK Biobank [27, 28], and we did not see any significant change in heart measures with ageing in the HD patients. This may be in part due to the fact that we excluded people with a history of IDH or severe heart failure and that the study population was relatively young (median age 59 years). The extent to which acute intradialytic changes in the brain contribute to patient-centred outcomes, in particular long-term cognitive impairment or chronic brain abnormalities demonstrable on imaging [5] is now being addressed through studies of cognitive testing, including the CONVINCE study [29]. Current studies employing MRI during or immediately after HD have not had adequate sample sizes to definitively study associations between acute WM changes and short-term alterations in cognitive function, nor have they included long-term follow-up with repeat imaging. Indirect evidence to support a causal link between subclinical ischaemia and the development of chronic WM lesions comes from a randomized trial of dialysate cooling, in which the intervention improved haemodynamic stability and protected against the development of WM abnormalities in DTI metrics [30], although acute changes in cerebral perfusion during dialysis were not measured. Our study identifies a potential new mechanism associated with non-haemodynamic, yet rather rapid osmotic changes, that may injure brain white matter. There are no equivalent studies on interventions that may influence osmotic solute and water shifts, but dialysis-based interventions are possible and warrant future study (e.g. moving to isotonic or isonatremic dialysate sodium, decreasing solute removal rates with more frequent dialysis schedules). In future, brain tissue sodium content during HD will be studied using MRI [31] and may provide valuable insights into osmotic and fluid shifts. This will be complemented by a detailed assessment of brain volume, DTI, perfusion and blood flow, aiming to identify potential intervention to reduce such brain injury.

Our study does have some weaknesses as it was dictated by the design of the HD-REMODEL trial [13], whose primary aim was to perform intradialytic MRI to study brain and cardiac haemodynamics. Due to the technical challenges of conducting intradialytic MRI, the sample size was relatively small, limiting the generalizability of our findings. We did not collect structural MPRAGE data and DTI data at multiple time points, precluding the assessment of GM/WM volume changes and FA/MD changes pre- to post-dialysis. The cognitive function tests measured in this study (TMT and MoCA) were not sensitive enough to allow comparison pre- and post-dialysis, and were within normal range limiting correlation with brain MRI measures. The short-term nature of the study precludes assessment of progression of WM abnormalities over time.

In conclusion, HD results in an increase in WM T_1_, reflecting an acute rise in brain water content independent from haemodynamic changes. Further research is required to determine how this relates to other acute changes in the brain during dialysis, to understand underlying mechanisms and whether this contributes to chronic pathological changes and accelerated brain ageing that is evident in this vulnerable patient group.

## Supplementary Material

gfaf117_Supplemental_File

## Data Availability

Please contact the corresponding author for data access.
