# Supplementary material for: Effect of haemodialysis on the brain and heart assessed using multiparametric MRI
Source: Nephrol Dial Transplant. 2025 Jul 9;40(12):2338–48. doi: 10.1093/ndt/gfaf117 (PMC12648064; doi:10.1093/ndt/gfaf117)
Supplement: gfaf117_Supplemental_File [file gfaf117_supplemental_file.docx]

**Supplementary Material**

Table of Contents:

Details of Cognitive Assessments

Detailed MRI Acquisition and Analysis

Details of additional healthy volunteer DTI datasets

Table S1: Relationship of brain MRI measures and cognitive test results with age and dialysis vintage in participants receiving haemodialysis (HD) and in healthy volunteers (HV).

Table S2: Relationship of heart MRI measures with age and dialysis vintage in participants receiving haemodialysis (HD) and in healthy volunteers (HV).

**Details of Cognitive Assessments**

Cognition was assessed using the Montreal Cognitive Assessment (MoCA) and the Trail Making Test (TMT). These are outlined in detail below.

MoCA: This test was originally developed as a tool for detecting early dementia in patients with mild cognitive complaints that perform in the normal range on the MMSE (Mini-Mental State Examination). It is a test that can be administered in 10 minutes and includes assessment of short-term memory recall, visuospatial abilities, executive functions, attention, concentration, working memory, language and orientation to time and place. The maximum score is 30.

TMT: This test provides an assessment of cognitive flexibility. There are 25 circles on a sheet of paper. In part A, the circles contain the numbers 1-25 and the participant is asked to use a pencil to connect the circles in numerical order. In part B, the circles contain the numbers 1-13 and letters A-L and the participant is asked to use a pencil to connect the circles alternately between numbers and letters in an ascending order (e.g. 1-A-2-B-3-C….). If the participant makes a mistake, they are informed and must correct the error. Before the participant attempts each part, the test part is demonstrated to them using a sample sheet. The time taken for each part to be completed correctly is noted. The average times for completion are 29 seconds and 75 seconds for parts A and B respectively.

**Detailed MRI Acquisition and Analysis**

**In a given scan session cardiac MRI measures were collected before brain MRI measures. The protocol comprised the following:**

*Measures of Cardiac Structure and Function (Scans 1-4, ~30 minute acquisition time)*

A left ventricular (LV) short axis cine scan was collected to assess left ventricular function and diastolic dysfunction. Cardiac-gated cine images at multiple time points across the cardiac cycle were collected with a multi-slice TFE (turbo field echo) sequence (FOV = 300 x 210 mm^2^, 12 slices, 30 phases, 3 slices acquired per 15-20 s expiration breath hold, slice thickness = 10 mm, reconstructed resolution = 1.17 x 1.17 mm, TR/TE = 3.9/1.94 ms, SENSE = 2). cine images were analysed using Philips Intellispace software (Philips Medical Systems). The user identified end diastole and end systole phases using all slices. Epicardial and endocardial contours were drawn on the end diastole image and propagated through all phases. This was repeated for all slices containing left ventricle blood pool. The basal slice was selected such that the end-diastole and end-systole images had at least fifty percent of the blood volume surrounded by myocardium. The apical slice was defined as the last slice showing an intra-cavity blood pool. The papillary muscles were included within the blood pool. The epicardial and endocardial contours are used together to calculate the wall mass, excluding the papillary muscles. The wall mass was adjusted for BSA to give LV wall mass index. For each cardiac phase, the end diastolic and end systolic blood volume contours were summed across slices to provide a graph of LV blood volume against time through the cardiac cycle. The end diastolic volume (EDV) and end systolic volume (ESV) were used to calculate stroke volume (SV = EDV – ESV) and cardiac output (CO = SV*heart rate). These were both indexed to a patient's body size by dividing by the body surface area (BSA) to compute stroke index and cardiac index. Ejection fraction was also calculated (EF = (EDV-ESV)/EDV).

T_1_ mapping data was collected using a Philips Cardiac Native 5(3)3 modified Look-Locker inversion recovery (MOLLI) scheme. Three slices through the short axis of the LV were acquired (base, mid, apex) with FOV = 320 x 320 mm^2^, reconstructed voxel = 0.9 x 0.9 mm^2^, slice thickness = 10 mm, 3 slices, SENSE = 2.5, FA = 20°, inversion delay time = 350 ms, TR/TE = 2.3/1.03 ms, 1 slice per 12 s breath hold. Analysis of MOLLI T_1_ data was performed using in-house Matlab (The MathWorks, Inc) software. Images were first assessed for motion, any data points with motion were discarded and then fit voxel-by-voxel to a Look-Locker inversion recovery to calculate a T_1_ map:

$Signal=abs\left( A-B.exp\left( -\frac{TI}{{T1}^{*}} \right) \right)$ where $T1\approx{T1}^{*}\left( \frac{B}{A}-1 \right)$

The goodness of fit was computed for each voxel and voxels with poor fits were removed from ROI analysis. A ROI was manually drawn to cover the myocardium using MIPAV v.8.0.2 software to obtain a median myocardial T_1_ value.

PC-MRI was used to assess aortic outflow (AO) with a single slice positioned across the ascending aorta with FOV = 280 x 264 mm^2^, TR/TE = 3.8/2.4 ms, FA = 15°, # averages = 3, reconstructed resolution = 0.97 x 0.97 x 10 mm^3^, velocity encoding = 300 cm/s, scan time was approximately 1 minute dependent on the patient’s heart rate. Data were analysed using Philips Viewforum Qflow software (Philips Medical Systems). A region of interest (ROI) was drawn around the vessel wall and propagated through all phases across the cardiac cycle. From this, waveforms of blood velocity, area and flux over the cardiac cycle were derived. The mean across the cardiac cycle of each parameter was calculated to obtain mean vessel cross-sectional area (mm^2^), mean blood velocity (cm/s), and mean flux of blood flow (ml/s) over the cardiac cycle. Cross sectional area and flux were adjusted to account for BSA.

Myocardial wall strain was assessed using MR tagging, which informs on how the heart contracts and twists between diastole and systole. TFE tagging data were acquired through both the long and short axis of the left ventricle. Parameters for the long axis were FOV = 350 x 350 mm^2^, reconstructed voxel = 0.9 x 0.9 mm^2^, slice thickness = 8 mm, SENSE 2, TR/TE = 5.7/3.5 ms, FA = 10°, grid spacing/angle = 7 mm/45°. Parameters for the short axis were FOV = 320 x 320 mm^2^, reconstructed voxel = 0.9 x 0.9 mm^2^, slice thickness = 10 mm, SENSE 2, TR/TE = 5.8/3.4 ms, FA = 10°, grid spacing/ angle = 7 mm/ 45°. Short axis tagging was acquired for 3 slices through the LV (base, mid, apex) matching the T_1_ mapping slices and the mid-slice matching the cardiac ASL perfusion measures. Tagging data was analysed using CIM v8.1 Tag2D (Auckland UniServices Ltd.) software. In both the long and short axis tagging the myocardial wall was defined by placing contours around the epicardium and endocardium on the second phase and then propagating the contours to the other phases. The tag was tracked through the cardiac phases to produce a strain graph from which the peak strain can be determined. Images where the tag had blurred due to poor breath holding were discarded from the analysis and only data where a peak was achieved was included. The strain was defined as longitudinal strain in the long axis and circumferential strain in the short axis. Longitudinal strain defines how the myocardium is compressed or stretched in the direction of the long axis. Circumferential strain describes the strain of the short axis as the myocardial wall is contracting inwards.

*Measures of Brain Volume and White Matter Tracts (Scan 1 only, ~6 minute acquisition time)*

A 3D T_1_-weighted MPRAGE image (1mm isotropic resolution; TR/TE = 8.3/3.8 ms, flip angle = 8°, SENSE factor = 3.5, 162 slices, 256 x 256 matrix) was acquired to assess grey and white matter volume and cortical thickness. Structural MPRAGE images were pre-processed using SPM12 [SPM12; Wellcome Department of Cognitive Neurology, University of London] and the associated toolbox CAT12 running on MATLAB [The MathWorks, Natick, MA] with default parameters given by the CAT12 toolbox. The T1-weighted MPRAGE data were segmented into grey matter volume (GM, white matter volume (WM), and cerebral spinal fluid (CSF) tissue classes and then transferred into the Montreal Neurological Institute (MNI) stereotactic coordinate space and normalized using a high- dimensional DARTEL normalization. The images were then smoothed with a Gaussian smoothing kernel of 12 mm (FWHM) to allow parametric comparisons across subjects. The final smoothed, modulated, and normalized images had a 1.5 mm isotropic voxel size.

Voxel-based morphometry statistical analysis was performed using a General Linear Model design including the total intracranial volume (TIV) as a regressor of no-interest to remove the effect of variation in brain size across participants from the data. Possible edge effects between tissue types were avoided by excluding all voxels with GM values of less than 0.1 (absolute threshold masking). The statistical threshold for assessing voxel-wise differences was set at [P<0.05, FDR corrected], cluster size >5. A multiple regression model was used to investigate the potential associations between GMV, WMV, and CSF with age and dialysis duration with results threshold at P<0.05, FDR corrected, cluster size > 5.

Diffusion Tensor Imaging (DTI) was used to assess the fractional anisotropy (FA) and mean diffusivity (MD). A 64 direction SE-EPI DTI sequence was collected with b-factor of 1000 s/mm^2^, (FOV = 224 x 224 mm^2^, reconstructed voxel size = 1.56 x 1.56 mm^2^, slice thickness 2.2 mm, 48 axial slices, SENSE = 1.3, Multiband factor = 3, half scan factor = 0.6, SPIR fat suppression, TR/TE = 2081/88 ms, EPI factor = 77, scan duration = 2 min 20 s). All images were analyzed using FSL5 software package (http://fsl.fmrib.ox.ac.uk/fsl). Images were realigned to remove eddy current distortions. Diffusion tensor elements were computed at each voxel using the FMRIB Diffusion Toolbox program by creating a brain mask using Brain Extraction Tool software and then fitting the diffusion tensor model to obtain fractional anisotropy (FA – a measure of the degree of anisotropy of a diffusion process) and mean diffusivity (MD – a measure of the magnitude of water diffusion within brain tissue) maps. All averages and SDs in FA and MD were extracted from images using fslmaths and fslstats programs.

*Measures of Brain Haemodynamics and Water content assessed by T_1_ Relaxometry (Scans 1-4, ~15 minute acquisition time)*

Cerebral blood flow of the right and left internal carotid artery flow and basilar artery flow was assessed using phase contrast MRI (PC-MRI). A single TFE slice was placed perpendicular to all three vessels with FOV = 150 x 101 mm^2^, reconstructed voxel size = 0.6 x 0.6 x 5 mm^3^, SENSE = 2, FA = 10°, TR/TE = 9.1/5.5 ms, half scan factor = 0.625, velocity encoding = 90 cm/s, scan duration = 39 s. Data were analysed using Philips Viewforum Qflow software (Philips Medical Systems). A region of interest (ROI) was drawn around the vessel wall and propagated through all phases across the cardiac cycle. From this, waveforms of blood velocity, area and flux over the cardiac cycle were derived. The mean across the cardiac cycle of each parameter was calculated to obtain mean vessel cross-sectional area (mm^2^), mean blood velocity (cm/s), and mean flux of blood flow (ml/s) through the internal carotid and basilar arteries.

To determine grey matter perfusion, Arterial Spin Labelling (ASL) data was acquired with a FAIR labelling scheme and SE-EPI readout. Imaging parameters were FOV = 240 x 240 mm^2^, reconstructed voxel size = 3 x 3 mm^2^, slice thickness = 6 mm, 10 axial slices, SENSE = 2.3, TR/TE = 4000/16 ms, EPI factor = 29, 30 pairs of selective/non-selective images, selective/non-selective thickness = 90/300 mm, label delay = 1500 ms, pre/post saturation, fat suppression = no, background suppression = no, vascular crushing = no, scan duration = 4 min 8 s. A Base equilibrium M0 scan with no labelling and inflow scan (20 pairs with label delay times = 300, 500, 800, 1200 ms) were also collected with the matched geometry and imaging parameters for perfusion quantification. A T_1_ mapping scheme with SE-EPI readout (TR = 8000ms) matched to the ASL acquisition and was also acquired with inversion times of 100, 200, 400, 600, 800, 1000, 1200, 1400, 2100, 3100 ms.

To assess brain T_1_ mapping measures, the inversion recovery data were fit on a voxel-by-voxel basis to a standard 2-parameter inversion recovery equation to generate T_1_ maps using in-house Matlab (The MathWorks, Inc) code. Partial volume (PV) maps of grey matter, white matter and cerebral spinal fluid (CSF) were created from the T_1_ maps (skull removed) using the FAST segmentation algorithm. Partial volume maps indicate how much of each voxel is comprised of each tissue type and is given as a fraction of 1. The PV maps were moved into MNI space to create common masks across all visits and scans for each of the subjects (these common masks are also used to assess grey matter perfusion). The common masks were transformed back into native space to interrogate the T_1_ maps. Two thresh holds were used (0.5, 0.75) to create two masks each for grey matter, white matter and CSF from the PV common masks. For each mask and tissue type, a histogram was plot and the mode and full width at half maximum (FWHM) calculated.

To analyse the perfusion data, all the selective and non-selective images (ASL and inflow) were realigned to the base M0 scan using MCFLIRT. Perfusion weighted difference maps were calculated for each selective/non-selective pair and then averaged to create a single perfusion-weighted (ΔM) map for each inversion time. The ΔM maps and base M0 were used to calculate perfusion maps using BASIL. For each subject, the grey matter common mask (obtained previously from the T_1_ maps) was then applied to the perfusion map at each time point with two thresholds (0.5, 0.75 as for the T_1_) and the median grey matter perfusion calculated at each threshold.

**Details of additional healthy volunteer DTI datasets**

Due to a technical problem, DTI data were only acquired in 4 of the HV subjects recruited for this study. We therefore included DTI data from 11 additional healthy subjects collected with an identical acquisition.

The graphs and table below give details comparing the original study group that had a DTI scan (green circles), with the additional subjects and combined altogether. There were no differences in age or DTI metrics of FA or MD between the original study group and the combined group (unpaired t-test, p=0.85, p=0.11, p=0.44 respectively).


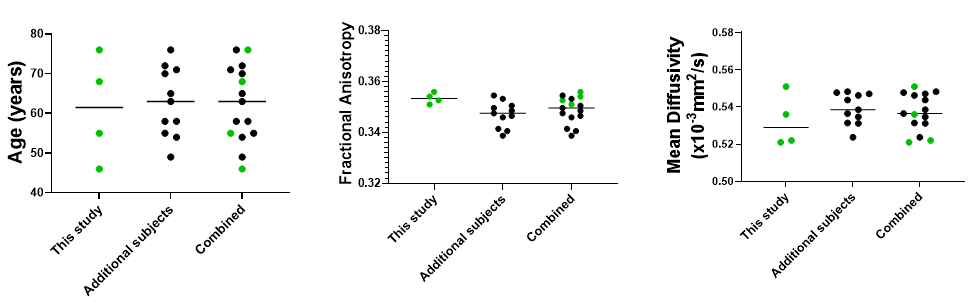


|  | **This Study** | **Additional subjects** | **Combined** |
| --- | --- | --- | --- |
| **Number of subjects with DTI data** | 4 | 11 | 15 |
| **Age (years)** | | | |
| Minimum | 46 | 49 | 46 |
| Maximum | 76 | 76 | 76 |
| Mean | 61 | 63 | 62 |
| Standard deviation | 13 | 9 | 10 |
| **Fractional Anisotropy** | | | |
| Minimum | 0.351 | 0.339 | 0.339 |
| Maximum | 0.356 | 0.355 | 0.356 |
| Mean | 0.353 | 0.347 | 0.349 |
| Standard deviation | 0.002 | 0.005 | 0.005 |
| **Mean Diffusivity (x10^-3^ mm^2^/s)** | | | |
| Minimum | 0.521 | 0.524 | 0.521 |
| Maximum | 0.551 | 0.548 | 0.551 |
| Mean | 0.533 | 0.539 | 0.537 |
| Standard deviation | 0.014 | 0.008 | 0.010 |

**Table S1: Relationship of brain MRI measures and cognitive test results with age and dialysis vintage in participants receiving haemodialysis (HD) and in healthy volunteers (HV).**

| Parameter | Multiple Linear Regression  with age and dialysis vintage | | | | Linear Regression  with age | | | | |
| --- | --- | --- | --- | --- | --- | --- | --- | --- | --- |
|  | **HD** | | | | | | **HV** | | **HD v HV** |
|  | **Goodness of fit: R^2^** | **ANOVA Overall Regression p -value** | **ANOVA parameter p-value**  **(sign of coefficient if significant)** | | **Goodness of fit: R^2^** | **Is the slope non-zero?**  **p-value (sign of coefficient if significant)** | **Goodness of fit: R^2^** | **Is the slope non-zero?**  **p-value (sign of coefficient if significant)** | **Are slopes significantly different?**  **P-value** |
|  |  |  | **Vintage** | **Age** |  |  |  |  |  |
| GMV/TIV | 0.2 | 0.3 | 0.8 | 0.3 | 0.2 | 0.1 | 0.4 | **0.03**  **(-)** | 0.7 |
| WMV/TIV | 0.5 | **0.04** | 0.5 | **0.07**  **(-)** | 0.5 | **0.01**  **(-)** | 0.5 | **0.01**  **(-)** | 0.1 |
| CSFV/TIV | 0.5 | 0.06 | 0.6 | 0.09 | 0.5 | **0.02**  **(+)** | 0.6 | **0.004**  **(+)** | 0.6 |
| GM T_1_ | 0.6 | **0.02** | 0.08 | 0.2 | 0.5 | **0.02**  **(+)** | 0.1 | 0.2 | 0.2 |
| WM T_1_ | 0.8 | **0.002** | **0.008**  **(+)** | 0.11 | 0.5 | **0.02**  **(+)** | 0.3 | **0.04**  **(+)** | 0.2 |
| MD | 0.7 | **0.02** | 0.4 | **0.03**  **(+)** | 0.7 | **0.005**  **(+)** | 0.3 | **0.03**  **(+)** | **0.003** |
| FA | 0.9 | **0.0007** | 0.4 | **0.0007 (-)** | 0.9 | **<0.0001**  **(-)** | 0.3 | **0.04**  **(-)** | **<0.0001** |
| MoCA Score | 0.3 | 0.3 | 0.7 | 0.2 | 0.2 | 0.1 | 0.01 | 0.7 | 0.3 |
| TMT Part A completion Time | 0.3 | 0.2 | 0.9 | 0.2 | 0.3 | 0.07 | 0.2 | 0.1 | 0.4 |
| TMT Part B completion Time | 0.4 | 0.1 | 0.9 | 0.1 | 0.4 | **0.03**  **(+)** | 0.7 | **0.001**  **(+)** | 0.6 |

*Abbreviations: GMV – grey matter volume, TIV – total intercranial volume, WMV – white matter volume, CSFV – cerebrospinal fluid volume, GM – grey matter, WM – white matter, MD – mean diffusivity, FA – fractional anisotropy, MoCA – Montreal Cognitive Assessment, TMT – Trail making test.*

**Table S2: Relationship of heart MRI measures with age and dialysis vintage in participants receiving haemodialysis (HD) and in healthy volunteers (HV).**

| Parameter | Multiple Linear Regression  with age and dialysis vintage | | | | Linear Regression  with age | | | | |
| --- | --- | --- | --- | --- | --- | --- | --- | --- | --- |
|  | **HD** | | | | | | **HV** | | **HD v HV** |
|  | **Goodness of fit: R^2^** | **ANOVA Overall Regression p -value** | **ANOVA parameter p-value**  **(sign of coefficient if significant)** | | **Goodness of fit: R^2^** | **Is the slope non-zero?**  **p-value (sign of coefficient if significant)** | **Goodness of fit: R^2^** | **Is the slope non-zero?**  **p-value (sign of coefficient if significant)** | **Are slopes significantly different?**  **P-value** |
|  |  |  | **Vintage** | **Age** |  |  |  |  |  |
| Ejection fraction | 0.07 | 0.7 | 0.4 | 0.8 | 0.004 | 0.8 | 0.01 | 0.7 | 0.99 |
| SV index | 0.04 | 0.8 | 0.9 | 0.7 | 0.04 | 0.5 | 0.5 | **0.006**  **(-)** | 0.1 |
| Cardiac index | 0.3 | 0.2 | 0.4 | 0.08 | 0.3 | 0.09 | 0.5 | **0.02**  **(-)** | 0.9 |
| Myocardial T1 | 0.2 | 0.5 | 0.8 | 0.4 | 0.1 | 0.2 | 0.007 | 0.8 | 0.3 |
| LV WMI | 0.2 | 0.4 | 0.3 | 0.9 | 0.05 | 0.5 | 0.02 | 0.7 | 0.4 |
| LA strain | N too small | | | | 0.06 | 0.6 | 0.2 | 0.99 | 0.1 |
| SA strain (apical slice) | 0.3 | 0.4 | 0.5 | 0.6 | 0.2 | 0.2 | 0.03 | 0.6 | 0.4 |
| SA strain (mid slice) | 0.2 | 0.6 | 0.4 | 0.98 | 0.06 | 0.5 | 0.3 | 0.09 | 0.9 |
| SA strain (basal slice) | N too small | | | | 0.2 | 0.3 | 0.1 | 0.3 | 0.2 |
| EDV/BSA | 0.07 | 0.7 | 0.6 | 0.4 | 0.05 | 0.5 | 0.6 | **0.006**  **(-)** | 0.3 |

*Abbreviations: SV – stroke volume, LV – left ventricle, WMI – wall mass index, LA – long axis, SA – short axis, EDV – end diastolic volume, BSA – body surface area.*
